# The Treatment of Acute Ileo-Colitis in Infancy and Childhood

**Published:** 1903-07

**Authors:** 


### Abstracts and Selections.

### The Treatment of Acute Ileo-Colitis in Infancy and Childhood.

In Dr. L. Emmett Holt's work upon "The Diseases of Infancy and Childhood," the second edition of which has recently been published, the following statement is made regarding the treatment of acute ileo colitis.

"As the pathological process is principally in the colon, and most severe in the lower half of the colon, it can often be much more effectually treated by injections than by drugs given by the mouth. Irrigation of the colon is one of our most valuable means of treatment in these cases. For general purposes, a saline solution at 100 to 104 degrees F. should be employed (common salt or borax, one drachm to water one pint). One or two quarts should be given at one time; it should be injected high into the colon through a long rectal tube, and early in the disease repeated at least twice a dav."

We are in full accord with the statement made by Dr. Holt that "irrigation of the colon is one of our most valuable means of treatment in these cases," but instead of employing a solution of salt or borax, we would suggest the use of Glyco-Thymoline, one ounce to the pint of water, which has proved very successful in the treatment of intestinal diseases of children. In Glyco-Thymoline we have an alkaline antiseptic solution, such as is indicated in these conditions, where we have to deal with an inflamed mucous membrane, with its acid secretions.

In a communication recently received from Geo. Howard Thompson, M. D., Professor of Materia Medica and Experimental Medicine at the St. Louis College of Physicians and Surgeons, he states: "This summer I have had a large number of cases of cholera infantum, subacute gastro-intestinal indigestion and marasmus. Probably my best results were derived from the following line of treatment:

The baby was given podophyllin, one-fortieth of a grain, or calomel, one-tenth of a grain, until the passages became distinctly bilious. Then the following intestinal antiseptic sedative and astringent was prescribed:

Bismuth subcarbonatis ......................dr. i
Glyco-Thymoline......................fl. oz. ss
Mistura cretæ....................q. s. ad. fl. oz. iii

M. Sig.—Shake the bottle and take a teaspoonful every three hours.

It is always a good idea to give the baby an enema of a pint of warm water containing an ounce of Glyco-Thymoline at the commencement of the treatment, in order that the colon may be relieved as rapidly as possible of its fermenting and decomposing contents, and that the mucous membrane of the gut may be brought in contact with the antiseptic and healing application. Daily enemata of the above solution are frequently advisable.

This summer I have had fewer unfortunate terminations in my cases of cholera-infantum than in previous years, and I attribute my good fortune largely to the fact that, at the instigation of Dr. A. E. Chatfield, of Cleveland, Ohio, I have added this non-irritating antiseptic Glyco-Thymoline to my internal medication in inflammatory diseases of the gastro-intestinal tract. It has proven to have these advantages: It is alkaline, it is antiseptic, it is non-toxic, it is non-irritating, it is healing. It is about the only antiseptic that can be safely given to children."

To supplement Dr. Thompson's flattering statement regarding the value of Glyco-Thymoline in the treatment of gastro-intestinal disorders in children, we will append the following brief clinical reports:

Fred P. Lowenstein, M. D., of Westfield, Mass., reports the case of a male child, three years old, who had been suffering from enterocolitis for five days, the attending physician having used without avail all the usual remedies. I was called to see the child in consultation. It was greatly emaciated, with an enlarged and tender abdomen. The temperature was 103.2 degrees F., but in spite of this the body was covered with cold perspiration. The stools were semi-solid, resembling chopped spinach in appearance. I advised warm applications of spices to the abdomen and ordered the following:

| | |
|---|---|
| Calcium carbon, precip | dr. ss |
| Tinc. opii. camp | dr. i |
| Glyco-Thymoline | oz. i |
| Aq. cinnamon | oz. ii |

A teaspoonful of this mixture was taken every four hours, and as a result the child had entirely recovered in three days.

A. D. Ellis, M. D., of Powersville, Mo., reports the following case: The patient, a child of sixteen months, had been suffering from vomiting and diarrhea for six days before I was summoned. The passages were foul and bloody. I immediately put the patient on Glyco-Thymoline and liquor bismuth, equal parts, a teaspoonful to be taken every two hours. Just after the second dose was given, I could see a marked change for the better. The patient improved rapidly and in about three days had entirely recovered. Another similar case, also treated with Glyco-Thymoline, gave me equally good results.

R. G. Waters, M. D., of Milam, Mo., reports the following case: The patient was a child, twenty months old, suffering from gastroenteritis, the vomiting being almost constant. I gave half a teaspoonful of Glyco-Thymoline in hot water every hour until five doses were taken, and also ordered an enema of Glyco-Thymoline, one tablespoonful to four ounces of water. This treatment gave prompt relief and I believe it saved the child's life.

